# Dietary Fiber Treatment Corrects the Composition of Gut Microbiota, Promotes SCFA Production, and Suppresses Colon Carcinogenesis

**DOI:** 10.3390/genes9020102

**Published:** 2018-02-16

**Authors:** Faraz Bishehsari, Phillip A. Engen, Nailliw Z. Preite, Yunus E. Tuncil, Ankur Naqib, Maliha Shaikh, Marco Rossi, Sherry Wilber, Stefan J. Green, Bruce R. Hamaker, Khashayarsha Khazaie, Robin M. Voigt, Christopher B. Forsyth, Ali Keshavarzian

**Affiliations:** 1 Department of Internal Medicine, Division of Gastroenterology, Rush University Medical Center, Chicago, IL 60612, USA; Phillip_Engen@rush.edu (P.A.E.); Nailliw_Preite@rush.edu (N.Z.P); Maliha_Shaikh@rush.edu (M.S.); Marco_Rossi@rush.edu (M.R.); Sherry_Wilber@rush.edu (S.W.); Robin_Voigt@rush.edu (R.M.V.); Christopher_Forsyth@rush.edu (C.B.F.); Ali_Keshavarzian@rush.edu (A.K.); 2 Whistler Center for Carbohydrate Research, Department of Food Science, Purdue University, West Lafayette, IN 47907-2009, USA; yunustuncil@odu.edu.tr (Y.E.T.); hamakerb@purdue.edu (B.R.H.); 3 DNA Services Facility, Research Resources Center, University of Illinois at Chicago, Chicago, IL 60612, USA; anaqib2@uic.edu (A.N.); greendna@uic.edu (S.J.G.); 4 Department of Biological Sciences, University of Illinois at Chicago, Chicago, IL 60607, USA; 5 Department of Immunology, Mayo Clinic College of Medicine, Mayo Clinic, Rochester, MN 55905, USA; Khazaie@mayo.edu; 6 Department of Physiology, Rush University Medical Center, Chicago, IL 60612, USA; 7 Division of Pharmacology, Utrecht Institute for Pharmaceutical Sciences, Utrecht University, 3584 CG Utrecht, The Netherlands; 8 Department of Pharmacology, Rush University Medical Center, Chicago, IL 60612, USA

**Keywords:** CRC, dietary fiber, microbiota, SCFA, butyrate

## Abstract

Epidemiological studies propose a protective role for dietary fiber in colon cancer (CRC). One possible mechanism of fiber is its fermentation property in the gut and ability to change microbiota composition and function. Here, we investigate the role of a dietary fiber mixture in polyposis and elucidate potential mechanisms using TS4Cre × cAPC^l^°^x468^ mice. Stool microbiota profiling was performed, while functional prediction was done using PICRUSt. Stool short-chain fatty acid (SCFA) metabolites were measured. Histone acetylation and expression of SCFA butyrate receptor were assessed. We found that SCFA-producing bacteria were lower in the polyposis mice, suggesting a decline in the fermentation product of dietary fibers with polyposis. Next, a high fiber diet was given to polyposis mice, which significantly increased SCFA-producing bacteria as well as SCFA levels. This was associated with an increase in SCFA butyrate receptor and a significant decrease in polyposis. In conclusion, we found polyposis to be associated with dysbiotic microbiota characterized by a decline in SCFA-producing bacteria, which was targetable by high fiber treatment, leading to an increase in SCFA levels and amelioration of polyposis. The prebiotic activity of fiber, promoting beneficial bacteria, could be the key mechanism for the protective effects of fiber on colon carcinogenesis. SCFA-promoting fermentable fibers are a promising dietary intervention to prevent CRC.

## 1. Introduction

Colon cancer (CRC) is the second leading cause of death from cancer. Although genetic susceptibility plays an important role in the pathogenesis of CRC, environmental factors play an even bigger role in the development of colon cancer. Indeed, multiple observational and epidemiological studies have demonstrated that CRC is closely associated with lifestyle and it is estimated that almost half of CRCs are preventable by a healthy lifestyle [1,2]. Among lifestyle-related factors, dietary habits are strongly associated with the disease risk. In particular, dietary fiber (i.e., cereal, fruit, and whole grains) has been shown to significantly modulate the CRC risk [3,4].

Fibers are indigestible in the gastrointestinal tract until arrival to the colon, where they are mainly fermented, resulting in the formation of short-chain fatty acids (SCFA) such as acetate, propionate, and butyrate (in a ratio of 3:1:1 approximately). Several anti-cancerous mechanisms have been shown for SCFAs, including their direct effects on cancerous epithelial cells, or anti-inflammatory effects in the colon as reviewed elsewhere [5,6].

Fermentation of the fiber in the colon is performed by the colonic microbiota. Therefore, in addition to the amount of fiber intake, varying microbiota may have differential effects on the fermentation ability of fiber, and could potentially modulate the risk of CRC. In this regard, several human studies have linked colonic microbiota to colon carcinogenesis [7,8]. However, despite relatively strong epidemiological data on fiber in CRC, results of experimental studies on polyposis models and CRC patients are controversial, and the main mechanisms that modulate the fiber effect have remained unclear [9,10,11].

The existing discrepancies could be partly explained by factors related to the type of animal model employed to represent human CRC, and/or considerations given to microbiota [12,13,14]. The type of fiber product under study could affect the outcome as the fiber structure can have a major impact on its function, including fermentation profile, as different groups of gut bacteria have different preferred substrates [15]. Thus, different fiber structure will favor/disfavor growth of different bacteria with different metabolites leading to different changes in the gut microenvironment, including intestinal epithelial cells. We hypothesized that fermentable fiber products that promote the production of SCFA have a beneficial effect on colon carcinogenesis. Here, we aimed to test our hypothesis by investigating the impact of a specific insoluble fermentable fiber mixture that has shown, in our in vitro model, to favor the production of SCFA, on colon carcinogenesis in a genetically susceptible host and elucidate potential mechanisms. To this end, we used a mouse polyposis model that occurs mainly in the colon where fermentation of fiber by microbiota occurs.

## 2. Materials and Methods

### 2.1. Animals

Animal experiments were carried out at Rush University Medical Center, Chicago, IL, USA, after approval of the animal protocol by the Institutional Animal Care and Use Committee at Rush (protocol number: 14-008, from: 02/01/2014). Mutations in the adenomatous polyposis coli (*APC*) gene are found in the majority of human CRC. To model CRC in this study, we chose TS4Cre × cAPC^l^°^×468^ mice (TS4/APC), which are double heterozygous mice with deletion of the adenomatous polyposis coli gene (APC^Δ468^) in the colonic epithelium. These TS4/APC mice are generated by crossing LoxP APC^Δ468^ mice with TS4Cre mice that harbor a *Cre* gene under the control of the fatty acid binding protein 1 (*FABP1*) gene promotor, restricted to the epithelial cells of the colon and terminal ileum, as previously described [16,17].

This study was divided into two phases that included male mice treated with only one type of diet or two different diets: Phase 1 utilized five C57BL/6J control mice (B6) (Jackson Laboratory, Bar Harbor, ME, USA) and five TS4/APC mice fed with a standard rodent chow diet (Teklad Envigo #2018, Madison, WI, USA) for 12 weeks. For Phase 2, only TS4/APC mice were used and randomly assigned to receive either the standard rodent chow diet (five mice) or Teklad Envigo #TD160445 high fiber diet (five mice). TD160445 is a modification of standard Envigo #2018 to include 20% insoluble fermentable fiber by weight—fiber included here consists of 50% chitin-glucan (KitoZyme SA, Herstal, Belgium) and 50% raw potato starch, prepared at Purdue University, West Lafayette, IN, USA, and shipped to Teklad for inclusion in the standard chow. Each treatment was given for 12 weeks.

For both study phases, age-matched four- to six-week-old male mice were housed individually in cages within ventilated light-tight cabinets. All animals were acclimated to the new housing for one week prior to initiating the study and maintained on a constant 12-h light/12-h dark cycle receiving food and water ad libitum for the duration of the experiment. Body weight and average food intake were not different between B6 and APC/TS4 mice (Figure S1). Similarly, for Phase 2 mice, where mice were fed with standard chow or the high fiber diet, body weight was not different between the two groups (Figure S2). After ten weeks of treatment, mice were placed in an empty non-bedded cage for 24 h and their stool was collected for microbial analysis. Mice were sacrificed by decapitation at week 12 and their intestinal tissue was collected; cecum tissue was stored at −80 °C for protein analysis, while the intestine was fixed in 10% buffered formalin for 24 h. After fixation, the number of polyps was counted for all the groups and the intestinal tissue was paraffin-embedded.

### 2.2. Histology and Tissue Staining

Paraffin-embedded large intestine tissue was cut in 5 μm sections and deparaffinized sections were stained with hematoxylin and eosin (H&E), as previously described [18,19]. Pictures of H&E stained slides of five mice per group were taken utilizing a Leica DMR microscope (Leica Microsystems, Wetzlar, Germany) and reviewed by a gastrointestinal pathologist, who blindly quantified the pictures for adenomas.

### 2.3. Western Blot

Cecum lysates of five mice per group were prepared utilizing modified cell lysis RIPA buffer (10 mM Tris, 200 mM NaCl, 5 mM EDTA, 1 mM PMSF and 10% Glycerin). Cold RIPA buffer containing 10% protease and 1% phosphatase inhibitor cocktails (Sigma-Aldrich, St. Louis, MO, USA) was added to 10 mg of cecum tissue. Samples were homogenized in buffer and lysates were centrifuged to remove any insoluble material. Supernatants were then assessed for total protein concentration using Pierce BCA Protein Assay Kit following the manufacturer’s instructions (ThermoFisher-Scientific, Waltham, MA, USA #23225).

Total protein (20 μg) was size fractionated via Western blot using 10% sodium dodecyl sulfate–polyacrylamide gel electrophoresis (SDS-PAGE), transferred to a nitrocellulose membrane and probed with GPR109a (Santa Cruz Biotechnology, Dallas, TX, USA #sc-134583—1:1000 dilution) and H3ac (EMD Millipore, Burlington, MA, USA #06-599—1:1000 dilution) rabbit polyclonal primary antibodies, and β-actin (Sigma-Aldrich, St. Louis, MO, USA #A2066- 1:5000 dilution) was used as a loading control. The secondary antibody solution used was anti-rabbit IgG conjugate HRP (Cell Signaling, Danvers, MA, USA #7074S), in a 1:2000 dilution. Western blot was performed using the Bio-Rad Mini-Protean 3 system (Bio-Rad, Hercules, CA, USA) and protein levels were scanned for densitometry analysis with NIH Image J software as described previously [20,21].

### 2.4. Microbiota Profiling and Bioinformatics Analysis

Total DNA was extracted from mice feces collected at ten weeks of experimental interventions utilizing a FastDNA bead-beating Spin Kit for Soil (MP Biomedicals, Solon, OH, USA) and processed using high-throughput Illumina amplicon sequencing, as described previously [22]. DNA from five mice per group was utilized: Phase 1 [TS4/APC (case) ad libitum chow + water male mice and B6 (control) ad libitum chow + water male mice feces]; Phase 2 [TS4/APC ad libitum chow + water male mice and TS4/APC ad libitum high fiber diet + water male mice]. Raw FASTQ files for each sample were merged using the software package PEAR (Paired-end-read merger) (v0.9.8) [23]. Merged reads were quality trimmed and sequences shorter than 250 bases were discarded (CLC Genomics Workbench, v10.0, CLC Bio, Qiagen, Boston, MA, USA). Sequences were screened for chimeras (USEARCH8.1 algorithm) [24], and putative chimeric sequences were removed from the dataset (QIIME v1.8) [25]. Each sample was rarefied and data were pooled, renamed, and clustered into operational taxonomic units (OTU) at 97% similarity (USEARCH8.1 algorithm). Representative sequences from each OTU were extracted and classified using the uclust consensus taxonomy assigner (Greengenes 13_8 reference database). A biological observation matrix (BIOM) [26] was generated at each taxonomic level from phylum to species (“make OTU table” algorithm) and analyzed and visualized using the software packages Primer7 [27] and the R programming environment [28].

Alpha diversity indices (within-sample) and Beta diversity (between-sample) were used to examine changes in microbial community structure between mice fecal group samples. Alpha diversity indices (i.e., Shannon, Simpson, richness, and evenness) were generated using the package ‘vegan’ implemented in the R programming language [29]. To examine differences in community composition between samples, a pairwise Bray–Curtis dissimilarity (non-phylogenetic) metric was generated using the Primer7 software package and used to perform analysis of similarity (ANOSIM) calculations. ANOSIM was performed at the taxonomic level of the genus, using square-root transformed data. The principal coordinates analysis (PCoA) method was used to visualize group overall microbial differences.

Differences in relative abundance of individual taxa, between mice fecal group samples, were assessed for significance using the Kruskal–Wallis test controlling for false-discovery rate (FDR), implemented within the software package QIIME [25]. Furthermore, mice fecal group sample’s community functional predictions were performed using PICRUSt (Phylogenetic Investigation of Communities by Reconstruction of Unobserved States) [30] and differences in Kyoto Encyclopedia of Genes and Genomes (KEGG) ortholog (KO) abundances between groups were identified [31].

Classification of putative “pro-inflammatory” and “anti-inflammatory” bacteria taxa was based on preceding reports [32,33,34,35,36]. Individual genus level putative SCFA-producing taxa were identified (acetate, propionate, or butyrate), their relative abundances summed up and total SCFA-producing bacteria differences between TS4/APC and B6 mice groups were calculated, using Mann–Whitney U (MWU) statistics [33,35,37,38,39,40,41,42,43].

### 2.5. SCFA Determination

Fecal samples of ten mice per group were collected after ten weeks of treatment and homogenized in carbonate–phosphate buffer (one part feces with three parts buffer). Samples were centrifuged at 13,000 rpm for 10 min. The supernatant (400 µL) was combined with 100 µL of internal standard (5% phosphoric acid—containing 50 mM of 4-methylvaleric acid and 8% of copper sulfate) and SCFA analysis was performed by gas chromatography equipped with a fused silica capillary column (NukonTM, Supelco No: 40369-03A, Bellefonte, PA, USA) and a flame ionization detector (GC-FID 7890A, Agilent Technologies, Inc., Santa Clara, CA, USA) under the conditions described previously [44]. SCFA quantification was assessed through measurements of the peak areas for acetate, propionate, and butyrate relative to 4-methyl valeric acid.

### 2.6. Statistical Methods

SPSS version 23 (SPSS, Inc., Chicago, IL, USA) was used for the statistical analyses. Proportions between categorical variables were compared between groups using the chi-square test or the Fisher’s exact test, where appropriate. Numeric results (polyp size and numbers) are presented as mean ± S.E.M., and were compared using two-tailed repeated measures analysis of variance (ANOVA) with Tukey’s multiple comparison test. A significance of *p*-value ≤ 0.05 was adopted. SAS 9.4 (SAS Institute Inc., Cary, NC, USA) was used for graph creation.

For Microbial Community Structure Analysis, in SPSS, two-tailed unpaired Independent t-test was used to analyze differences for parametric data satisfying test assumptions and Mann–Whitney U (MWU) was used to analyze nonparametric data. Correlations between SCFA concentrations and bacterial taxa were analyzed by Pearson’s correlation analysis. Alpha-diversity indices were calculated such as Shannon index (H′ = −∑ sum(Pi/log(Pi)) where Pi = the relative abundance of each taxon), Pielou’s evenness (J′ = H′/log(S)) where S = number of taxa present in each sample), richness (number of taxa present in each sample), and Simpson’s index (D = ∑(Pi2)) where Pi = the relative abundance of each taxon. Graphs were created using GraphPad Prism (v5.00) software.

## 3. Results

### 3.1. Polyposis Is Associated with Change in Fecal Microbiota

Five TS4/APC mice (case) and five B6 mice (control) were kept in single cages and fed a similar diet in Phase 1. Food (Figure S1) and water intake were similar between the groups. After 12 weeks, all the animals were sacrificed. Similar ages of cases and controls were selected to avoid age-specific microbiota changes in rodents [45]. As expected, some cases developed polyps while none of the controls had polyps.

**Figure 1:**
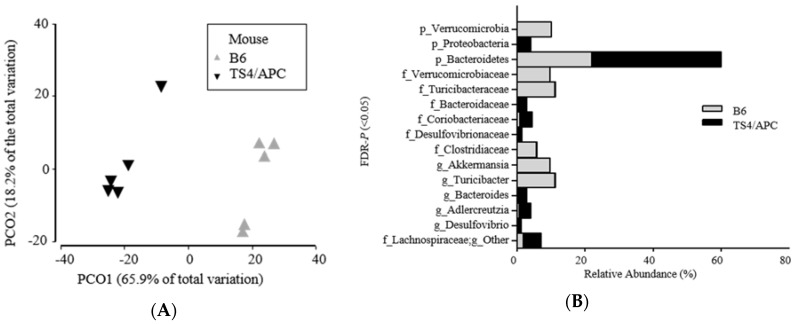
(**A**,**B**) Significantly different microbial profiles between TS4/APC (TS4Cre × cAPC^l^°^×468^ mice) chow-fed and B6 (C57BL/6J control mice) chow-fed mice feces: (**A**) Principal coordinate analysis (PCoA) profile of microbial diversity (genus) across all samples, with the percentage of variation explained by PC1 and PC2 indicated in the axis; (**B**) Stacked bars of significantly different (FDR-*p* ˂ 0.05) rarefied sequences of microbial taxa (relative abundance percentages of total bacteria at phylum, family, and genus) between mice groups.

Next, we compared microbiota composition between the two groups (TS4/APC and B6 mice). Stool was collected two weeks prior to the sacrifice to capture changes in the microbiota. The fecal microbiota analysis indicated that TS4/APC mice had increased alpha diversity in richness (*p* = 0.014), compared to the B6 (control), at the genus level. Upon analyzing beta diversity, the overall microbial community analysis revealed a significant difference between mice groups at the taxonomic level of the genus (ANOSIM: Global *R* = 1.00; *p* = 0.008) (Figure 1A). Figure 1B depicts the relative abundance of three phyla, six families and six genera that were significantly different (FDR-*p* ˂ 0.05) between the TS4/APC and B6 mice.

**Figure 2:**
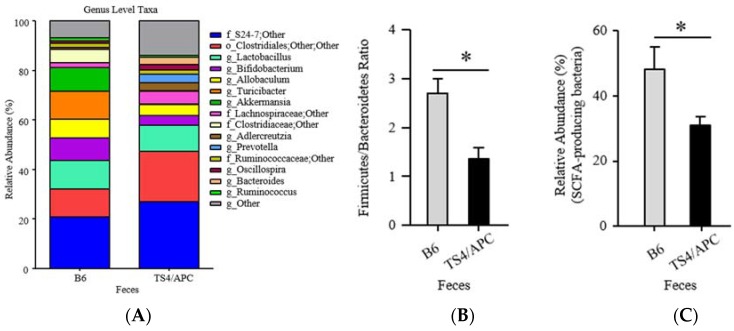
(**A–C**) Significantly different microbial profiles between TS4/APC and B6 chow-fed mice feces: (**A**) Stacked histograms depicting 90% of the rarefied sequences of microbial taxa (relative abundance percentages of total bacteria at the genus) between mice groups; (**B**) Firmicutes-to-Bacteroidetes ratio and; (**C**) Short-chain-fatty-acid (SCFA) bacterial taxa both significantly lower in TS4/APC chow-fed mice.

At the phylum level, Bacteroidetes and Proteobacteria were higher in TS4/APC mice, while Verrucomicrobia was increased in B6 mice. At the genus taxonomic level, *Bacteroides* (Gram-negative, bacteria), *Adlercreutzia* (equol-producing bacteria), and *Desulfovibrio* (pro-inflammatory/sulfate-reducing bacteria) were significantly increased in the TS4/APC mice [37]. It should be noted that *Desulfovibrio* colonize, utilize and degrade the colonic mucin layer, and could induce barrier dysfunction [46], a contributing phenomenon to CRC [47]. Increase in *Desulfovibrio* has been recently reported in another animal model of CRC [48]. Finally, Figure 2A indicates the genus level taxa that contribute to 90% of the microbial data between mice groups.

The ratio of Firmicutes-to-Bacteroidetes (F/B) was significantly different between groups (MWU; *p* = 0.015), indicating a decrease in F/B in the TS4/APC mice (Figure 2B). A decreased F/B ratio has been shown to predict a decrease in SCFA production [49,50]. Assessment of the relative abundance of the genus taxonomic level of SCFA-producing taxa confirmed that TS4/APC mice had a significant decrease in total SCFA-producing bacteria (MWU; *p* ˂ 0.05) (refer to methods), compared to the B6 mice (Figure 2C).

Overall, these findings suggested a potential decline in the fermentation product of dietary fibers in polyposis mice and extended a possible preventive role for interventions that target the colon microbiota functionality and SCFA production [51].

### 3.2. High Fiber Treatment Alters the Microbiota Composition in Polyposis Mice

Early experiments showed that dietary modification could affect the gut environment and SCFA levels in the digestive tract [52]. Since the discovery of the critical role of gut microbiota in the pathogenesis of multiple intestinal and systemic diseases including CRC [53,54,55], several gut microbiota-directed interventions have been employed to change the gut microenvironment towards a healthier environment [56]. Here, we treated polyposis mice with our high fiber mixture designed to favor the growth of SCFA-producing bacteria to modulate the intestinal microbiota and consequently modulate levels of colonic SCFA.

**Figure 3:**
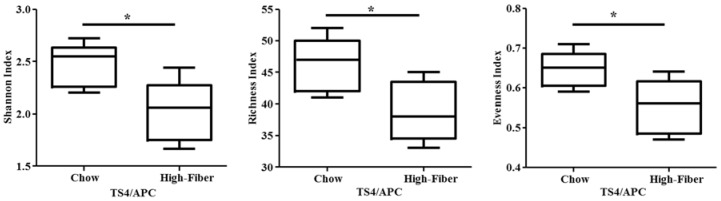
Alpha diversity is significantly lower in TS4/APC high fiber-fed mice compared to chow-fed mice. Plots show the Shannon (*p* = 0.023), Richness (*p* = 0.033), and Evenness (*p* = 0.033) values between mice groups at the genus level. Two-tailed unpaired Independent *t*-test was used to analyze differences for parametric data satisfying test assumptions.

Polyposis mice (TS4/APC) were treated with chow-based diet supplemented with or without the high fiber mixture for 12 weeks. Fiber supplementation changed the microbiota composition significantly with a significant increase in SCFA-producing bacteria. The fecal microbiota analysis showed that TS4/APC high fiber-fed mice had decreased alpha diversity, compared to the TS4/APC chow-fed mice (Figure 3).

**Figure 4:**
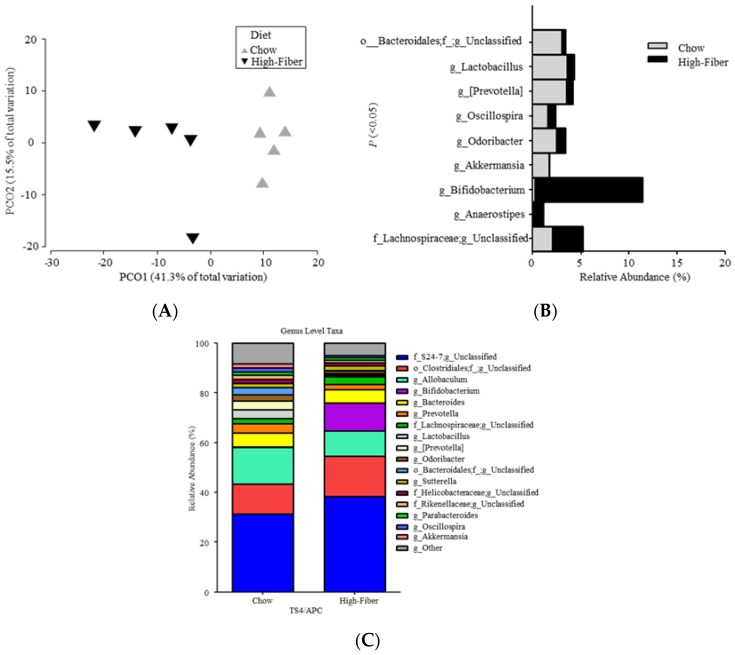
(**A–C**) Significantly different microbial profiles between TS4/APC chow-fed and high fiber-fed mice feces: (**A**) Principal coordinate analysis (PCoA) profile of microbial diversity (genus) across all samples, with the percentage of variation explained by PC1 and PC2 indicated in the axis; (**B**) Stacked bars of significantly different (*p* ˂ 0.05) rarefied sequences of microbial taxa (relative abundance percentages of total bacteria at genus) between mice groups; (**C**) Stacked histograms depicting 90% of the rarefied sequences of microbial taxa (relative abundance percentages of total bacteria at the genus) between mice groups.

When analyzing beta diversity, the F/B ratio showed no change between chow-fed and high fiber-fed mice (MWU; *p* = 1.00). Significant overall microbial community structure differences, between chow-fed and high fiber-fed mice, were observed at the taxonomic level of the genus (ANOSIM: Global *R* = 0.74; *p* = 0.008) (Figure 4A). Furthermore, the relative abundance of nine genera indicated significant differences between chow-fed and high fiber-fed mice (Figure 4B).

When compared to chow-fed mice, the high fiber-fed mice had significant increases in putative SCFA-producing bacteria *Bifidobacterium, Lachnospiraceae*_unclassified, and *Anaerostipes* [37]. Figure 4C compares sequences of microbial taxa (relative abundance percentages of total bacteria at the genus) between the mice groups.

**Figure 5:**
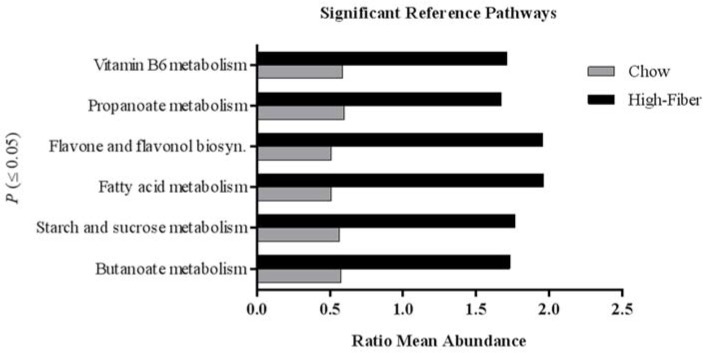
Functional predictions for fecal microbiomes of TS4/APC high fiber-fed and chow-fed mice. Significantly different KEGG (Kyoto Encyclopedia of Genes and Genomes) pathways (*p* < 0.05) with a greater ratio mean abundance in TS4/APC high fiber-fed mice relative to chow-fed mice, as inferred using PICRUSt (Phylogenetic Investigation of Communities by Reconstruction of Unobserved States) analysis of fecal microbiomes.

Using a predicative assessment of the microbial community functional potential (PICRUSt), with a specific a priori hypothesis of examining SCFA-related pathways, we identified six pathways and 30 KOs that had significantly increased abundances in high fiber-fed mice compared to chow-fed mice (Figure 5) (Tables S1 and S2). Particularly, SCFA butanoate and propanoate metabolism pathways were increased in high fiber-fed mice. Given the pattern of the changes in the microbiota, next we assessed the SCFA metabolites affected by the high fiber treatment.

### 3.3. High Fiber Treatment Increased the Short Chain Fatty Acid Production in Polyposis Mice

Change in the microbiota composition has been shown to alter the bacterial metabolites in mice [57]. We measured the effect of the high fiber treatment on SCFA metabolites in feces by a Nukol fused silica capillary column (see method).

**Figure 6:**
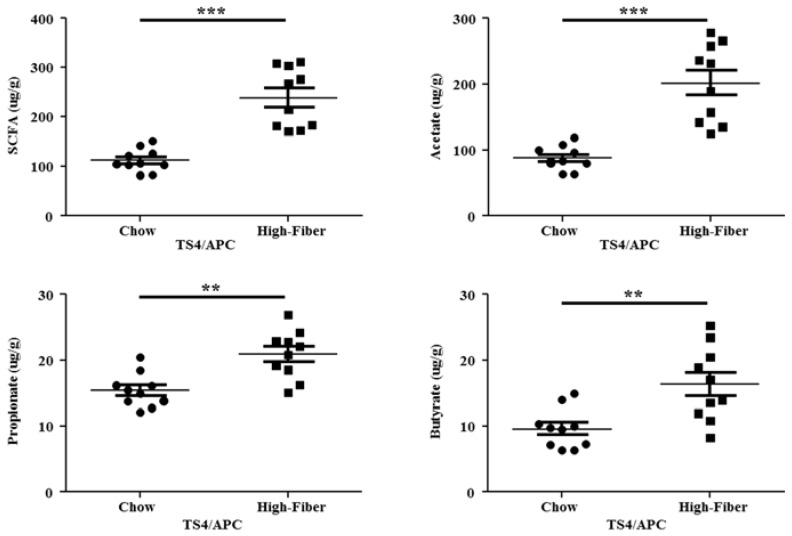
Significant changes in all short-chain fatty acids’ (SCFAs) metabolite levels, when examined in the entire cohort of TS4/APC chow-fed and high fiber-fed mice. Total SCFA concentration (MWU; *p* ˂ 0.0001), acetic acid (MWU; *p* ˂ 0.0001), propionic acid (*p* = 0.0011), and butyric acid (*p* = 0.0034) were all significantly increased in fecal samples of high fiber-fed mice compared to chow-fed mice. Mice (*n* = 10) per group.

Within the high fiber-fed mice fecal samples, a significant increase in the total SCFA concentration (*p* = 0.0025) and each SCFA separately were detected, compared to chow-fed mice (Figure 6).

We then correlated the microbial composition with SCFA metabolites. We observed a significant positive correlation between high fiber-fed mice and the genera *Bifidobacterium* (p_Actinobacteria), *Lachnospiraceae*_unclassified (p_Firmicutes) and *S24-7*_unclassified (p_Bacteroidetes) (Table 1). The positive correlations were as follows: (*Bifidobacterium*: total SCFA and acetate); (*Lachnospiraceae*_unclassified: total SCFA, acetate, and butyrate); (*S24-7*_unclassified: propionate). Genera that negatively correlated with high fiber-fed chow mice were *Prevotella* (p_Bacteroidetes), [*Prevotella*] (p_Bacteroidetes) and *Lactobacillus* (p_Firmicutes). Significant changes in all SCFA metabolites and microbiota are listed in Table 1.

### 3.4. Increased Short Chain Fatty Acid Is Associated with Improved Polyposis

**Figure 7:**
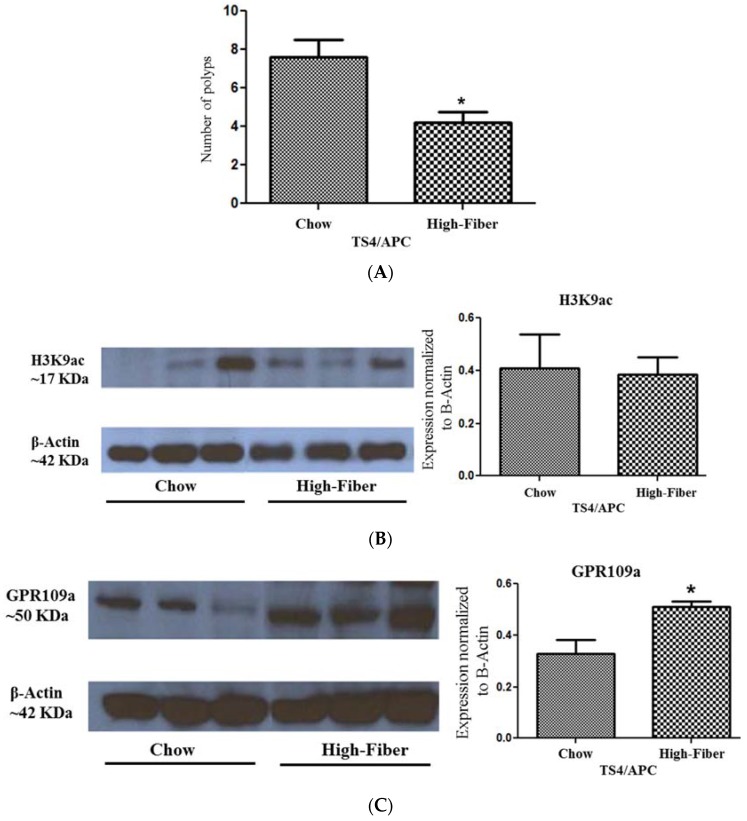
(**A–C**) Effect of the high fiber diet on polyposis in TS4/APC mice (*n* = 5 per group): (**A**) Number of polyps was significantly reduced in high fiber-fed mice compared to chow-fed mice; (**B**) Left: Representative of histone acetylation blot, as assessed by H3K9ac antibody, on cecum tissue of high fiber-fed and chow-fed mice; Right: Western blot Expression of H3K9ac was normalized to the expression of Actin and was compared between the two groups; (**C**) Left: Representative of GPR109a blot using cecum tissue of high fiber-fed and chow-fed mice; Right: Western blot Expression of GPR109a, after normalization to Actin expression, increased by fiber treatment (*n* = 5 per group); asterisk represents *p* < 0.05.

Higher abundance of specific taxa capable of SCFA production prior to polyposis has been shown to be associated with decreased tumor burden in rodents [58]. Here, we investigated the effect of increase in SCFA due to high fiber treatment on polyposis. A comparison of tumor numbers of the two groups showed a significant decrease in polyposis in high fiber-treated mice, which had a lower number of polyps compared to untreated mice (Figure 7A).

SCFAs can exert their effects on colon carcinogenesis through two major pathways—signaling through their receptors or intracellular distribution—resulting in histone deacetylase inhibition (HDACi) [6,59,60]; these pathways are shown to result in mucosal integrity, epithelium apoptosis, and repression of pro-tumorigenic inflammation [61,62].

In order to determine the main potential molecular pathway underlying the observed protective effect of SCFA-promoting fiber on polyposis, we compared the histone acetylation levels as an estimate of histone deacetylase inhibitor (HDACi) capacity of SCFA, in high fiber-treated and untreated mice. Histone acetylation, as estimated by the expression level of PanH3ac (Histone H3ac pan-acetyl), an estimate of HDACi capacity between high fiber and chow-fed mice, was not affected by fiber treatment (Figure 7B), making HDACi less likely to be involved in the fiber effect in our model, in spite of the fact that high fiber treatment increased the butyrate level, which has been widely accepted as the most potent HDAC inhibitor responsible for the beneficial effects of SCFA in CRC [38,39,40,41,42,43,44,45,46,47,48,49,50,51,52,53,54,55,56,57,58,59,60,61,62,63].

We then compared the expression of the butyrate-specific receptor, GPR109a [64], in high fiber-treated and untreated animals. We observed an increased GPR109a level in high fiber-treated mice (Figure 7C), associated with ameliorated polyposis, consistent with GPR109a as the mediating signal delivering the effect of microbiota metabolite butyrate to offset colonic tumorigenesis [64,65].

## 4. Discussion

Colon cancer (CRC) is closely linked to lifestyle-associated factors including diet. Dietary fiber has been shown to reduce CRC risk by several epidemiological studies [3,4]. Fermentation of dietary fiber by colon microbiota forms SCFAs which are proposed to deliver anti-cancerous capacities of dietary fiber [5,6]. Here, we show that polyposis mice harbor “dysbiotic” microbiota, characterized by a decrease in the relative abundance of SCFA-reducing bacteria. High fiber supplementation changes microbiota population significantly, increases SCFA-producing bacteria, augments SCFA-related functional pathways, and elevates the levels of SCFA metabolites. The increase in SCFA was associated with a significant decrease in tumor load.

The APC mutation and loss of function of the APC gene product is an early and frequent event in human CRC. APC(+/Min) models in rodents show extensive polyposis which almost ubiquitously involves the small bowel, whereas adenoma or adenocarcinoma in human small bowel is a very rare event. Here, we model human CRC using TS4/APC mice that develop polyps mainly in the large bowel. TS4/APC animals are created from the C57BL/6J (B6) strain. In our study, the initial comparison of microbiota was made between these two groups from the same strain.

Firmicutes and Bacteroidetes are the two major bacterial phyla that comprise the majority of the normal gut microbiota of healthy humans. Proteobacteria and Actinobacteria constitute the remaining 10% of the microbial system [66]. Similarly, in mice, Firmicutes and Bacteroidetes represent the main taxa, while Proteobacteria is among the major remaining phyla. Polyposis mice had increased relative abundance of Proteobacteria phyla, which has been associated with both colonic adenomas and colorectal cancer [67]. We found an association between tumor occurrence and the relative abundance increase in Bacteroidetes and decrease in the relative abundance of Firmicutes and Bacteroidetes (F/B ratio) in the TS4/APC mice, when compared to B6 mice where no polyps developed. Altered microbial abundances of Firmicutes and Bacteroidetes are shown to affect the risk of CRC in humans, depending on the subclusters of these phyla [68]. For example, the species *Bacteroides fragilis* that belongs to Bacteroidetes has been repeatedly shown to be associated with colorectal neoplasms and to promote CRC [69]. We also observed an increase in the relative abundance of genus *Bacteroides* in TS4/APC mice. Additionally, within our study, the genus *Desulfovibrio* was associated with polyposis. *Desulfovibrio* is a sulfate-reducing bacteria with pro-inflammatory capacities [37]. *Desulfovibrio* can colonize, utilize and degrade the colonic mucin layer, and could induce barrier dysfunction [46], a phenomenon contributing to CRC [47]. Increase in the relative abundance of *Desulfovibrio* has been recently reported in another animal model of CRC [48].

An altered ratio of Firmicutes and Bacteroidetes has been associated with a variety of pathologies associated with aging [49]. In an APC-based mouse model of polyposis, an increase in the F/B ratio was associated with a decrease in tumor load [70]. In the model of TS4/APC specifically, our mouse group reported a decreased F/B ratio in association with worsening polyposis [22]. Evidence of a decreased Firmicutes to Bacteroidetes ratio has been linked with colonic inflammation in individuals with inflammatory bowel disease [71], and helps predict a decrease in the relative abundance of SCFA production [49,50]. We confirmed this pattern by finding a decrease in SCFA-producing taxa in TS4/APC compared to B6 mice.

SCFAs are the main metabolites of microbial fermentation of fiber, a potential protective diet against CRC according to epidemiologic studies. Based on reduced SCFA-producing bacteria and the potential decline in the fermentation product of dietary fibers in polyposis mice, we targeted microbiota composition by feeding polyposis mice with a high fiber regimen. This intervention increased SCFA-producing bacteria in polyposis mice. Particularly, the high fiber treatment significantly increased the relative abundances in both acetate-producing bacteria *Bifidobacterium* and both butyrate-producing clostridial cluster XIVa bacteria *Lachnospiraceae* unclassified and *Anaerostipes*. These three putative beneficial gut microbial taxa have been shown to be diminished in colorectal cancer [72]. *Bifidobacteria* is important to gut health as it has been shown to inhibit enteropathogens and promote gut health by interacting with butyrate-producing bacteria by cross-feeding interactions within the colon [39,73,74]. *Bifidobacterium* has also exhibited protected effects in several cancer cell lines [75]. Additionally, *Lachnospiraceae* unclassified and *Anaerostipes* are believed to benefit the host’s gut barrier functions through anti-inflammation, anti-tumorigenesis, and pathogen exclusion in the colon [39,76,77].

Increase in SCFA-producing bacteria resulted in the augmentation of butanoate and propanoate metabolism pathways upon high fiber treatment in our study. Direct measurement of SCFA metabolites confirmed the increase in SCFAs. A high fiber diet in humans has been shown to be associated with improved levels of SCFAs and reduced overall risk of CRC [78]. An increase in dietary fiber has resulted in an increase in butyrogenesis in a human trial [79]. An increase in SCFAs and butyrate from high fiber treatment in our study was associated with the amelioration of polyposis. These findings are consistent with a previous experiment, where increased SCFA abundance resulted in a reduction of pre-cancerous lesions in mice [80], and are in line with prior studies showing that SCFAs protect against the development of CRC, with most studies focusing on colonic butyrate production’s key role in CRC prevention [32].

Several mechanisms have been proposed to underlie the anti-cancerous mechanisms of SCFAs [5,6]. SCFAs, particularly butyrate, can inhibit histone deacetylases (HDACs). The increased histone acetylation modulates the transcriptional activities of several tumor suppressors as well as immune modulatory genes that could result in the down-regulation of inflammation and CRC risk [32,33,34,35,36,37,38,39,40,41,42,43,44,45,46,47,48,49,50,51,52,53,54,55,56,57,58,59,60,61,62,63,64,65,66,67,68,69,70,71,72,73,74,75,76,77,78,79,80,81]. However, we found no difference in PanH3ac expression, an estimate of HDACi capacity between high fiber-fed and chow-fed mice. In addition to epigenetic mechanisms, butyrate can exert its effect via GPR receptors. Fiber treatment and improved polyposis, in our model, were associated with an increased GPR109a level. GPR109a is a butyrate-specific receptor [64] that is lost during colonic neoplasms [82]. Recovery of GPR109a function is shown to increase the apoptotic and antiproliferative properties of CRC cells [64]. More recently, it was found that in addition to direct epithelial effects, GPR109a could modulate immune-epithelial cross talk in the colon, and when lost, further fuel colon carcinogenesis via increased pro-tumorigenic colonic inflammation [65].

## 5. Conclusions

Our data, along with other accumulating evidence, suggests fiber intervention as an effective and safe approach for microbiota reprograming to reduce colon tumorigenesis [83]. Our findings indicate that insoluble fermentable fiber increases putative SCFA-producing bacteria, SCFA-related functional pathways, SCFA metabolites, and positively correlates with putative beneficial microbial taxa. Correction of dysbiosis and improved polyposis upon fiber supplementation in our study is in line with recent data showing that the protection of dietary fibers could be microbiota dependent [84], and supports an observation in a human study where intestinal microbiota were found to mediate the association between fiber and CRC [85].

In summary, we showed that favorable effects of microbiota-directed intervention on polyposis are likely mediated via gut microbial functionality, mainly through SCFA production [51]. Overall, our results suggest a protective role of insoluble fermentable fiber in colon cancer, and provide a strong scientific rationale for future clinical trials to determine the therapeutic impact of SCFA-promoting fiber to prevent colon polyp/CRC.

## Figures and Tables

**Table 1:** Significant correlations comparing microbiota and SCFA concentrations shown by TS4/APC ad libitum high fiber-fed mice.

Taxa	Total SCFA v. High Fiber	Acetate v. High Fiber	Propionate v. High Fiber	Butyrate v. High Fiber
	*R, p*-Value	*R, p*-Value	*R, p*-Value	*R, p*-Value
Phylum				
Actinobacteria	+0.88, ˂0.00	+0.91, ˂0.00	-	-
Family				
Bifidobacteriaceae	+0.89, ˂0.00	+0.91, ˂0.00	-	-
Prevotellaceae	−0.80, ˂0.00	−0.78, ˂0.00	-	−0.73, 0.01
[Paraprevotellaceae]	−0.71, 0.02	−0.67, 0.03	-	−0.65, 0.04
Lactobacillaceae	−0.73, 0.02	−0.71, 0.02	-	-
o_RF32;f_unclassified	−0.65, 0.04	−0.65, 0.04	-	-
Mycoplasmataceae	−0.65, 0.04	-	-	-
S24-7	-	-	+0.66, 0.03	-
o_Streptophyta; f_unclassified	-	-	−0.77, 0.01	−0.82, ˂0.00
Genus				
*Bifidobacterium*	+0.89, ˂0.00	+0.91, ˂0.00	-	-
*Prevotella*	−0.80, ˂0.00	−0.78, ˂0.01	-	−0.66, 0.03
*[Prevotella]*	−0.73, 0.02	−0.70, 0.02	-	-
*Lactobacillus*	−0.73, 0.02	−0.71, 0.02	-	-
*f_Lachnospiraceae*; *g*_unclassified	+0.66, 0.03	+0.65, 0.04	-	+0.63, 0.04
*Coprococcus*	−0.66, 0.03	−0.68, 0.02	-	-
*f_Erysipelotrichaceae*; *g*_unclassified	−0.74, 0.01	−0.71, 0.02	-	-
*o_RF32*; *f*_unclassified; *g*_unclassified	−0.65, 0.04	−0.65, 0.04	-	-
*f_Mycoplasmataceae*; *g*_unclassified	−0.65, 0.04	-	-	-
*S24-7;g*_unclassified	-	-	+0.66, 0.03	-
*o_Streptophyta*; *f*_unclassifed; *g*_unclassified	-	-	−0.77, 0.01	−0.82, ˂0.00

*R* = Pearson *R* value; *p*-Value = (*p* < 0.05).

## Supplementary Materials

The following are available online at http://www.mdpi.com/2073-4425/9/2/102/s1, Table S1: List of significantly different KEGG pathways with greater abundance in TS4/APC ad libitum high fiber-fed mice relative to TS4/APC ad libitum chow-fed mice, as inferred using PICRUSt analysis of fecal microbiomes, Table S2: Select list of significantly different abundant KO’s between TS4/APC ad libitum high fiber-fed mice relative to TS4/APC ad libitum chow-fed mice, as inferred using PICRUSt analysis of fecal microbiomes, Figure S1 shows the body weight and average food intake of B6 and APC/TS4 mice, and Figure S2 shows a body weight comparison of APC/TS4 mice fed standard chow or the modified high fiber diet.
