# Supplementary material for: Dietary Fiber Treatment Corrects the Composition of Gut Microbiota, Promotes SCFA Production, and Suppresses Colon Carcinogenesis
Source: Genes (Basel). 2018 Feb 16;9(2):102. doi: 10.3390/genes9020102 (PMC5852598; doi:10.3390/genes9020102)
Supplement: Supplementary file 1 [file genes-09-00102-s001.docx]

**Supplementary Tables:**

**Table S1.** List of significantly different KEGG pathways with greater abundance in TS4/APC ad libitum high fiber-fed mice relative to TS4/APC ad libitum chow-fed mice, as inferred using PICRUSt analysis of fecal microbiomes.

| **PATHWAY** | **Abundance Mean, High-Fiber** | **Abundance Mean, Chow** | **High-Fiber/Chow Ratio** | ***p-*Value^a^** |
| --- | --- | --- | --- | --- |
| Butanoate metabolism | 97,519.80 | 56,336.00 | 1.73 | 0.02 |
| Starch and sucrose metabolism | 157,814.20 | 89,339.20 | 1.77 | 0.02 |
| Fatty acid metabolism | 36,561.00 | 18,653.20 | 1.96 | 0.04 |
| Flavone and flavonol biosynthesis | 1,919.20 | 980.60 | 1.95 | 0.04 |
| Propanoate metabolism | 73,214.80 | 43,862.40 | 1.67 | 0.04 |
| Vitamin B6 metabolism | 34,742.40 | 20,341.40 | 1.71 | 0.04 |
| *p*-value^a^ = *p* < 0.05: Kruskal-Wallis non-parametric one-way analysis of variance. | | | | |

**Table S2.** Select list of significantly different abundant KO’s between TS4/APC ad libitum high fiber-fed mice relative to TS4/APC ad libitum chow-fed mice, as inferred using PICRUSt analysis of fecal microbiomes.

| **Pathway or KEGG Ortholog** | **KEGG Ortholog** | **Abundance Mean, High-Fiber** | **Abundance Mean, Chow** | **High-Fiber/Chow Ratio** | ***p*-Value^a^** |
| --- | --- | --- | --- | --- | --- |
| Butanoate metabolism | | | | | |
| K00656 | formate C-acetyltransferase [EC:2.3.1.54] | 12,117.60 | 6,697.20 | 1.81 | 0.02 |
| K01652 | acetolactate synthase I/II/III large subunit [EC:2.2.1.6] | 9,759.00 | 5,010.60 | 1.95 | 0.01 |
| K01653 | acetolactate synthase I/III small subunit [EC:2.2.1.6] | 7,902.00 | 4,179.80 | 1.89 | 0.02 |
| K04072 | acetaldehyde dehydrogenase / alcohol dehydrogenase [EC:1.2.1.10 1.1.1.1] | 4,252.00 | 1,369.00 | 3.11 | 0.02 |
| K07250 | 4-aminobutyrate aminotransferase / (S)-3-amino-2-methylpropionate transaminase [EC:2.6.1.19 2.6.1.22] | 1,814.60 | 539.40 | 3.36 | 0.01 |
| Starch and sucrose metabolism | | | | | |
| K00700 | 1,4-alpha-glucan branching enzyme [EC:2.4.1.18] | 8,704.20 | 4,919.80 | 1.77 | 0.02 |
| K00703 | starch synthase [EC:2.4.1.21] | 7,321.00 | 4,397.20 | 1.66 | 0.04 |
| K00845 | glucokinase [EC:2.7.1.2] | 13,605.60 | 8,520.80 | 1.60 | 0.04 |
| K00847 | fructokinase [EC:2.7.1.4] | 7,192.80 | 4,078.60 | 1.76 | 0.04 |
| K00975 | glucose-1-phosphate adenylyltransferase [EC:2.7.7.27] | 8,505.60 | 4,303.00 | 1.98 | 0.04 |
| K01182 | oligo-1,6-glucosidase [EC:3.2.1.10] | 3,239.00 | 1,284.60 | 2.52 | 0.02 |
| K01188 | beta-glucosidase [EC:3.2.1.21] | 6,059.60 | 3,664.80 | 1.65 | 0.04 |
| K01193 | beta-fructofuranosidase [EC:3.2.1.26] | 4,456.80 | 1,606.20 | 2.77 | 0.01 |
| K02438 | glycogen debranching enzyme [EC:3.2.1.196] | 4,310.00 | 1,176.80 | 3.66 | 0.02 |
| K05349 | beta-glucosidase [EC:3.2.1.21] | 24,198.20 | 12,903.80 | 1.88 | 0.04 |
| K05350 | beta-glucosidase [EC:3.2.1.21] | 2,070.80 | 487.40 | 4.25 | 0.01 |
| Fatty acid metabolism | | | | | |
| K00645 | [acyl-carrier-protein] S-malonyltransferase [EC:2.3.1.39] | 6,135.40 | 3,930.20 | 1.56 | 0.04 |
| K00648 | 3-oxoacyl-[acyl-carrier-protein] synthase III [EC:2.3.1.180] | 6,504.80 | 4,349.20 | 1.50 | 0.04 |
| K01897 | long-chain acyl-CoA synthetase [EC:6.2.1.3] | 13,764.60 | 6,601.00 | 2.09 | 0.04 |
| K01961 | acetyl-CoA carboxylase, biotin carboxylase subunit [EC:6.4.1.2 6.3.4.14] | 7,467.40 | 5,041.40 | 1.48 | 0.04 |
| Flavone and flavonol biosynthesis | | | | | |
| K01195 | beta-glucuronidase [EC:3.2.1.31] | 1,919.20 | 980.60 | 1.96 | 0.04 |
| Propanoate metabolism | | | | | |
| K00048 | lactaldehyde reductase [EC:1.1.1.77] | 3637.00 | 1727.40 | 2.11 | 0.02 |
| K00382 | dihydrolipoamide dehydrogenase [EC:1.8.1.4] | 6321.20 | 3552.40 | 1.78 | 0.04 |
| K00656 | formate C-acetyltransferase [EC:2.3.1.54] | 12117.60 | 6697.20 | 1.81 | 0.02 |
| K01734 | methylglyoxal synthase [EC:4.2.3.3] | 3640.00 | 2027.20 | 1.80 | 0.02 |
| K01961 | acetyl-CoA carboxylase, biotin carboxylase subunit [EC:6.4.1.2 6.3.4.14] | 7467.40 | 5041.40 | 1.48 | 0.04 |
| K07250 | 4-aminobutyrate aminotransferase / (S)-3-amino-2-methylpropionate transaminase [EC:2.6.1.19 2.6.1.22] | 1814.60 | 539.40 | 3.36 | 0.01 |
| Vitamin B6 metabolism | | | | | |
| K00868 | pyridoxine kinase [EC:2.7.1.35] | 3,198.00 | 1,519.00 | 2.11 | 0.02 |
| K06215 | pyridoxal 5'-phosphate synthase pdxS subunit [EC:4.3.3.6] | 2,850.00 | 917.40 | 3.11 | 0.01 |
| K08681 | 5'-phosphate synthase pdxT subunit [EC:4.3.3.6] | 2,807.80 | 846.40 | 3.32 | 0.01 |

**Supplementary Figures:**

| 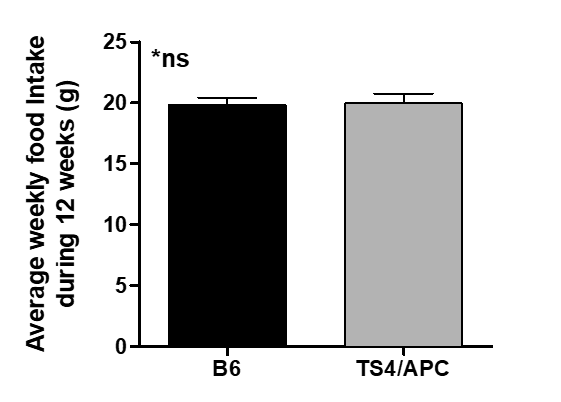  (**A**) | 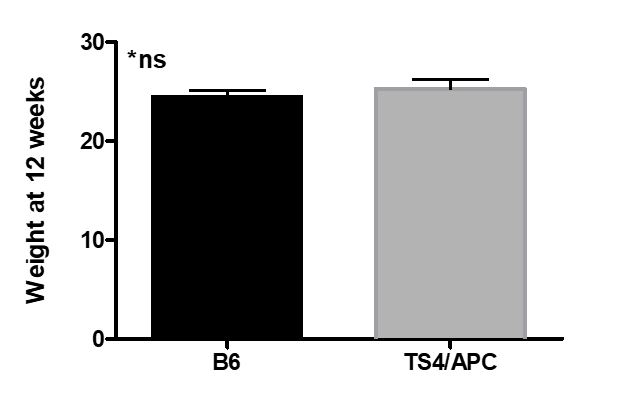  (**B**) |
| --- | --- |

**Supplementary Figure 1 (A-B).** Comparison of food intake and body weight between B6 and TS4/APC mice: (**A**) Average food intake and; (**B**) body weight in B6 and TS4/APC mice (*ns: *p* value is non-significant).


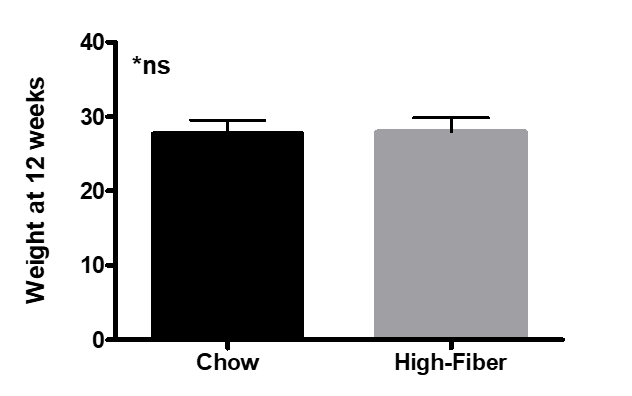


**Supplementary Figure 2.** Comparison of the body weight between TS4/APC fed by standard chow or high-fiber diet (*ns: *p* value is non-significant).
